# Forensic psychiatry in the age of the internet: the use of internet forums in the promotion and planning of an adolescent mass shooting

**DOI:** 10.47626/2237-6089-2021-0414

**Published:** 2023-08-04

**Authors:** Lisieux Elaine de Borba Telles, Thiago Henrique Roza, Saulo Maia Martins da Silva, Máira Oliveira Bitencourt, Caio Gibaile Soares Silva, Bibiana de Borba Telles, Victor Mardini, Alcina Juliana Soares Barros

**Affiliations:** 1 Departamento de Psiquiatria e Medicina Legal Universidade Federal do Rio Grande do Sul Porto Alegre RS Brazil Programa de Pós-Graduação em Psiquiatria e Ciências do Comportamento, Departamento de Psiquiatria e Medicina Legal, Universidade Federal do Rio Grande do Sul (UFRGS), Porto Alegre, RS, Brazil.; 2 Hospital de Clínicas de Porto Alegre Porto Alegre RS Brazil Serviço de Psiquiatria de Adições e Forense, Hospital de Clínicas de Porto Alegre (HCPA), Porto Alegre, RS, Brazil.; 3 Centro de Pesquisa Experimental Centro de Pesquisa Clínica HCPA Porto Alegre RS Brazil Laboratório de Psiquiatria Molecular, Centro de Pesquisa Experimental (CPE) e Centro de Pesquisa Clínica (CPC), HCPA, Porto Alegre, RS, Brazil.; 4 Instituto Nacional de Ciência e Tecnologia Translacional em Medicina Porto Alegre RS Brazil Instituto Nacional de Ciência e Tecnologia Translacional em Medicina (INCT-TM), Porto Alegre, RS, Brazil.; 5 HCPA Porto Alegre RS Brazil Serviço de Psiquiatria da Infância e Adolescência, HCPA, Porto Alegre, RS, Brazil.; 6 Universidade Federal de Ciências da Saúde de Porto Alegre Porto Alegre RS Brazil Universidade Federal de Ciências da Saúde de Porto Alegre (UFCSPA), Porto Alegre, RS, Brazil.

Recently, an editorial written by Watts et al. discussed the use of machine learning algorithms in the prediction of criminal offenses in psychiatric patients, with a focus on the debate about the ethical implications and the risks of stigmatizing individuals through the use of these promising tools in practical settings.^1^ This discussion is timely and of growing importance. However, the current situation in Brazil is that forensic psychiatric settings still need to adapt to relatively older technology, which concerns the expression of criminal and psychopathological behavior on the internet.

The internet, and the anonymity it may offer, has facilitated planning and execution of several different criminal behaviors, which may range from online stalking,^2^ dissemination of child pornography and other forms of illegal pornography,^3^ to the sharing of hate speech, extremist manifestos and terrorist propaganda.^4 , 5^ Nonetheless, health professionals in the Brazilian context, including forensic psychiatrists, hardly ever investigate such suspicious activities when assessing an individual patient. Therefore, in this letter, we aim to call attention to a relatively rare phenomenon in Brazil, which is promotion and planning of mass shootings on online forums, illustrating this discussion with the description of an individual case.

Here we report the case of a 15-year-old male, who, at the time of assessment, was an elementary school student in the 9th grade. He was referred to psychiatric inpatient care after the mother of a friend who he invited to take part in the attack discovered (ten days prior to the scheduled date) that he was planning a mass shooting in his own school and also intended to take his own life afterwards. He reported that he had carefully planned the act for approximately one year, learning about methods, specific clothing, and weapons on internet forums, on which he exchanged technical information with other individuals interested in similar events. He also planned the attack to take place on the same day as another Brazilian mass shooting, which had happened at a school in Suzano, São Paulo, one year earlier. During evaluation, the patient presented advanced technical knowledge about previous mass shootings such as those at “Columbine” and “Virginia Tech,” mentioning being an admirer of the perpetrators and also expressing the desire to be equally recognized. In the psychiatric assessment, the patient presented antisocial and narcissistic personality traits including: low frustration tolerance, lack of empathy, attitude of superiority, unusual interest in violence, sadistic behavior towards his 4-year-old brother, recurrent use of lies, periodic theft of money from family members, misuse of alcohol and marijuana, and also frequently skipping classes at school. In the year prior to his psychiatric hospitalization, he showed signs of depression and suicidal ideation, also presenting a history of cutting. Psychotic symptoms and mania/hypomania were not reported. After hospital discharge, he received outpatient psychiatric treatment for several months, until being discharged to a primary care institution. The patient and both of his parents signed consent for this case report to be written and published.

Mass shootings are a subtype of “mass murders,” in which three or more individuals are killed in a specific incident involving use of firearms, excluding the death of the perpetrator from the count.^6^ Adolescent mass shootings are considered to be a subtype of this phenomenon and the aggressor is usually a male adolescent acting on his own.^7^ Even though there are considerable discrepancies regarding descriptive statistics of mass shootings,^6^ there has been a significant increase in the incidence of these events in the US over the last 30 years.^8^ Usually, the perpetrators of these attacks present suicidal intentions and a desire for fame and perceive themselves as having suffered victimization by others, frequently using this as justification for the attacks.^9^ Beyond the incident-related casualties, mass shootings also have the potential to cause long-term effects in exposed individuals, being linked to depression, posttraumatic stress disorder (PTSD), and anxiety symptoms.^10^

In most cases, mass shootings are carefully premeditated attacks with intensive and long term planning that may take months or even years, with a focus on increasing the lethality of such acts.^9^ In the case reported here, the patient used online forums for this stage of planning, with the use of such websites also potentially having stimulated him into considering the attack in the first place. The role of social media and internet forums in similar events has been reported previously in the press. For instance, in the “Christchurch” attack in New Zealand, the perpetrator streamed his massacre in real time on Facebook, and posted racist messages on Twitter and 8chan prior to the event; the latter is an online forum previously associated with the dissemination of hate speech, conspiracy-theories and extremist manifestos.^11 , 12^ Minutes before the attack, the shooter in the “El Paso” incident posted a document filled with hate messages on the same 8chan website.^11 , 12^ Additionally, there has been a debate about the potential role of the media in promoting generalized imitation of this behavior. The extensive and detailed media coverage in the aftermath of a mass shooting can potentially act as a source of technical information concerning the methods used, as well as unwillingly promoting the social status of the perpetrator, which may be desired by other potential attackers as means of recognition.^13^ These potential indirect effects of the media coverage can be seen in the case described here as well.

Therefore, this case illustrates the potential use of internet forums in the promotion and planning of mass shootings. Fortunately, in this specific context, the adolescent’s intentions were not carried out because his plan was leaked before the scheduled date. Brazilian healthcare professionals and law enforcement agents should be aware of these internet tools and make use of them to identify potential perpetrators and prevent future attacks.
